# Comparative transcriptomic analysis reveals the regulatory mechanism of the gibberellic acid pathway of Tartary buckwheat (*Fagopyrum tataricum* (L.) Gaertn.) dwarf mutants

**DOI:** 10.1186/s12870-021-02978-8

**Published:** 2021-04-30

**Authors:** Zhaoxia Sun, Xinfang Wang, Ronghua Liu, Wei Du, Mingchuan Ma, Yuanhuai Han, Hongying Li, Longlong Liu, Siyu Hou

**Affiliations:** 1 College of Agriculture, Institute of Agricultural Bioengineering, Shanxi Agricultural University, Taigu, 030801 Shanxi China; 2 Key Laboratory of Crop Gene Resources and Germplasm Enhancement on Loess Plateau, Ministry of Agriculture, Taiyuan, 030031 Shanxi China; 3 Center for Agricultural Genetic Resources Research, Shanxi Agricultural University, Taiyuan, 030031 Shanxi China; 4 Shanxi Key Laboratory of Minor Crop Germplasm Innovation and Molecular Breeding, Taiyuan, 030031 Shanxi China

**Keywords:** Tartary buckwheat, Dwarf mutant, Transcriptome, Phytohormone

## Abstract

**Background:**

Tartary buckwheat is an important minor crop species with high nutritional and medicinal value and is widely planted worldwide. Cultivated Tartary buckwheat plants are tall and have hollow stems that lodge easily, which severely affects their yield and hinders the development of the Tartary buckwheat industry.

**Methods:**

Heifeng No. 1 seeds were treated with ethylmethanesulfonate (EMS) to generate a mutant library. The dwarf mutant *ftdm* was selected from the mutagenized population, and the agronomic characteristics giving rise to the dwarf phenotype were evaluated. Ultra-fast liquid chromatography-electrospray ionization tandem mass spectrometry (UFLC-ESI–MS/MS) was performed to determine the factors underlying the different phenotypes between the wild-type (WT) and *ftdm* plants. In addition, RNA sequencing (RNA-seq) was performed via the HiSeq 2000 platform, and the resulting transcriptomic data were analysed to identify differentially expressed genes (DEGs). Single-nucleotide polymorphism (SNP) variant analysis revealed possible sites associated with dwarfism. The expression levels of the potential DEGs between the WT and *ftdm* mutant were then measured via qRT-PCR and fragments per kilobase of transcript per million mapped reads (FPKM).

**Result:**

The plant height (PH) of the *ftdm* mutant decreased to 42% of that of the WT, and compared with the WT, the mutant and had a higher breaking force (BF) and lower lodging index (LI). Lower GA4 and GA7 contents and higher contents of jasmonic acid (JA), salicylic acid (SA) and brassinolactone (BR) were detected in the stems of the *ftdm* mutant compared with the WT. Exogenous application of GAs could not revert the dwarfism of the *ftdm* mutant. On the basis of the transcriptomic analysis, 146 homozygous SNP loci were identified. In total, 12 DEGs with nonsynonymous mutations were ultimately identified, which were considered potential candidate genes related to the dwarf trait. When the sequences of eight genes whose expression was downregulated and four genes whose expression was upregulated were compared, SKIP14, an F-box protein whose sequence is 85% homologous to that of SLY1 in Arabidopsis, presented an amino acid change (from Ser to Asn) and was expressed at a lower level in the stems of the *ftdm* mutant compared with the WT. Hence, we speculated that this amino acid change in SKIP14 resulted in a disruption in GA signal transduction, indirectly decreasing the GA content and downregulating the expression of genes involved in GA biosynthesis or the GA response. Further studies are needed to determine the molecular basis underlying the dwarf phenotype of the *ftdm* mutant.

**Conclusion:**

We report a Tartary buckwheat EMS dwarf mutant, *ftdm*, suitable for high-density planting and commercial farming. A significant decrease in GA4 and GA7 levels was detected in the *ftdm* mutant, and 12 DEGs expressed in the stems of the *ftdm* mutant were selected as candidates of the dwarfing gene. One nonsynonymous mutation was detected in the *SKIP14* gene in the *ftdm* mutant, and this gene had a lower transcript level compared with that in the WT.

**Supplementary Information:**

The online version contains supplementary material available at 10.1186/s12870-021-02978-8.

## Background

Tartary buckwheat (*Fagopyrum tataricum* (L.) Gaertn, Polygonaceae), an annual herbaceous crop plant species, is grown worldwide [1, 2]. Tartary buckwheat can grow in infertile soils and is adapted to both arid and semi-arid land. This species is well suited for cultivation in remote mountainous areas in China, which is important for potential alleviation of poverty. Compared with common buckwheat, Tartary buckwheat, which contains natural bioactive flavonoids, has high nutritional and utilization value; moreover, the latter also contains 10- to 100-fold higher levels of rutin [3–5]. This compound is used as a drug for reducing blood sugar, blood fat, and cholesterol; softening blood vessels; reducing capillary fragility; and preventing both skin cancer and Alzheimer’s disease [6, 7].

With the increasing demand for industrial buckwheat products, new buckwheat cultivars suitable for mechanized harvesting are needed. Tartary buckwheat plants are tall and have very brittle hollow stems; therefore, these plants are susceptible to lodging and bending, resulting difficulties during harvest that directly reduce production [8, 9]. Thus, it is important to improve the yield of Tartary buckwheat by breeding for shorter, stronger plants that resist lodging.

In the 1960s, the large-scale application of IR8, the first semi-dwarf rice mutant, greatly increased rice yields and ushered in the Green Revolution of agriculture [10]. Since the 1960s, plant height (PH) has become one of the most important targets for modern crop breeding because research has shown that introduced semi-dwarf varieties of cereal crop species present increased harvest indexes and increased yields, partially due to improved lodging resistance [11]. Rice (*sd1*) and wheat (*Rht*) dwarf mutants perform well under dense planting conditions, are resistant to lodging, and present increased yields [12, 13]. The mechanism underlying dwarfing in plants is complex: hormone synthesis, signal transduction, and gene regulation are be related to dwarfing [14]. There are many anatomical and physiological changes involved in plant dwarfism, such as shortened internode length (IL), decreased internode numbers, abnormal cell wall or cell elongation, and differences in plant hormone synthesis or signalling [15, 16]. In-depth research on plant dwarfing serves as a basis for identifying genes that govern excellent plant traits and for applying these genes to molecular breeding. Studies have shown that alterations to plant hormone signalling pathways are important causes of dwarfing. Both the reduced height (*Rht*) gene in wheat and the *sd1* gene in rice inhibit the gibberellin (GA) signal transduction pathway, resulting in reduced endogenous GA synthesis. In addition, previous studies have found that auxin (IAA), brassinosteroids (BRs) and strigolactone (SL) are related to plant dwarfing [17–19].

**Fig. 1:**
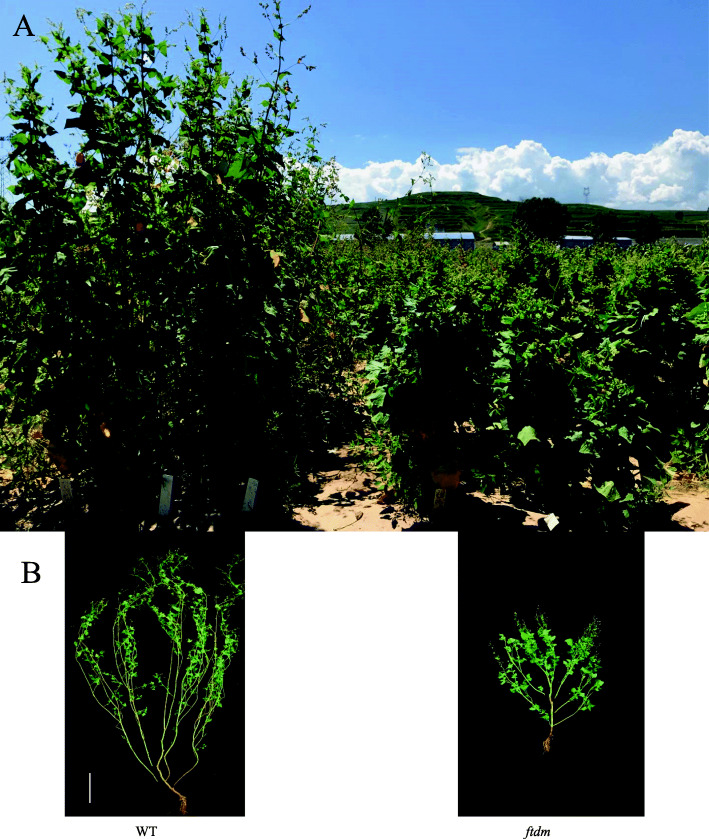
The phenotype of WT and *ftdm*. **a** The WT(left) and *ftdm* mutant (right) of buckwheat plants growing in the field. **b** The aboveground plant parts were imaged in the laboratory. The images were collected at Taigu, Shanxi, China, in August 2019. The bar on the left in **a** and **b** indicates a PH of 20 cm.

We obtained a dwarf mutant line (*ftdm*) from the cultivar Heifeng No. 1 by large-scale screening of an ethylmethanesulfonate (EMS)-mutagenized population. Through systematic breeding for many years, the PH was reduced from 160 to 210 cm for the wild type (WT) to 60–80 cm for the mutant (Fig. 1). Typically, buckwheat is grown 20 cm apart in rows, with 15 cm between rows. Assuming ~ 60,000 and ~ 80,000 WT and dwarf mutants seedlings/667 m^2^, respectively, we can calculate the volume rate to reflect the potential productivity. The volume rate of the WT was 0.011 (square metres/individual plants = 667 m^2^/60,000), which contrasted with 0.0083 (667 m^2^/80,000) for the dwarf mutant. This planting density and the lodging resistance of the dwarf mutant not only increased the number of plants in the population but also increased potential buckwheat yields. The dwarf mutant can be used as a genetic resource for future dwarf breeding, as well as in studies on hormone synthesis, other metabolic pathways, cytology, and the molecular mechanism underlying dwarfing. In addition, the availability of this dwarf mutant can make important contributions to promoting the development of the Tartary buckwheat industry.

## Methods

### Plant materials, growth conditions, and measurements of agronomic traits

The cultivar Heifeng No. 1 (WT line) was chosen for construction of the mutant library. The breeding process is summarized as follows: 1) Ten thousand seeds were soaked in 1.2% EMS solution overnight (10–12 h) and then neutralized in 1% Na_2_S_2_O_3_ for 5 min (repeated 3–5 times). The seeds were subsequently air dried under a fume hood and then immediately sown in the field. In 2014, we harvested M1 seeds and obtained the first mutant library. We then planted the seeds composing the library of selected individual dwarf lines from M2-M6. In 2019, a genetically stable dwarf mutant line, *ftdm,* was obtained. Afterward, *ftdm* and WT lines were grown in the field at Shanxi Agricultural University’s (northern China, 37°25′N, 112°29′E) experimental station during the summers from 2014 to 2019, and all the agronomic trait data were measured in 2019. One hundred plants from each line were planted in 2 m long rows spaced 0.3 m wide. Ten individuals of both the *ftdm* and WT lines were used to measure the following agronomic traits: PH (cm), IL (cm), stem diameter (SD; mm), and 1000-grain weight (1000-GW; g). The PH at 43 days after sowing (DAS; vigorous growth stage), 65 DAS (early flowering stage), and 145 DAS (maturity) was measured using a metric ruler (minimum range = 0.01 m). The fresh weight (FW, g) and root:shoot ratio (RS ratio) were determined at harvest.

### Determination of the lodging resistance index and stem strength indicator

Stem lodging of Tartary buckwheat typically occurs at the lower internodes (third (N3) or fifth (N5) internode from bottom). The number of internodes was determined according to the order from the bottom to the top of the plant. The breaking force (BF) of the N3 and N5 internodes was measured with a plant digital force gauge (YYD-1, Tuopuyunong Co., China); five replications were included for each trait. The distance between the fulcra of the tester was set to 10 cm. The lodging index (LI) was calculated according to the formula published by Zhang et al. [20]:

$$ \mathrm{LI}=\frac{\mathrm{FW}\left(\mathrm{g}\right)\times \mathrm{PH}\left(\mathrm{cm}\right)}{\mathrm{BF}\times 10\ } $$

### Cell tissue sections and microscopy

The N3 internode of the stem was cut into 0.5 cm^2^ slices that were fixed overnight in formalin–acetic acid–alcohol (FAA) solution (5 mL of formalin, 5 mL of acetic acid, and 90 mL of 70% alcohol) in a bottle. The slices were then dehydrated in a graded ethanol series (50, 70, 95, and 100%) before being embedded in paraffin (58–60 °C). After the samples dried for 2 ~ 3 days at 37 °C, transverse and longitudinal sections were cut from the embedded blocks. The slices were subsequently stained with 0.5% Fast Green (Sigma-Aldrich, Shanghai, China) for 30–60 s at room temperature, after which images were captured using a microscope (Olympus, Japan).

### RNA extraction and RNA sequencing (RNA-seq)

The N3 stem internodes were sampled from three *ftdm* and WT individuals at full bloom, pooled and then used for RNA extraction and RNA-seq analysis. Three individual biological replications were included for RNA-seq. Total RNA was extracted using TRIzol reagent (Invitrogen, USA) and then treated with DNase I (Thermo Scientific, USA). Six separate cDNA libraries were constructed, each with a 300 bp fragment insert size. Quality control checks for all the libraries were performed with an Agilent 2100 Bioanalyzer system according to the manufacturer’s protocol. The qualified cDNA libraries were ultimately sequenced by an Illumina HiSeq 2000 instrument (Gene Denovo, Guangzhou, China), and 150 bp paired-reads were generated. Clean reads were obtained by removing adapters, reads containing poly-N and low-quality reads, were then mapped to the tartary buckwheat genome (download from www.mbkbase.org/Pinku1) using Hisat2 [21]. After mapping clean reads onto reference genome, the gene expression levels were quantified with HTseq [22].

### Identification of single-nucleotide polymorphisms (SNPs) and functional annotations

To identify the SNPs from the RNA-seq data, we used GATK to recognize single-base mismatches between sequencing samples and the reference genome as potential SNP loci. Further, these SNP loci were queried via BLAST to identify their location in the genome and then annotated using the non-redundant nucleotide database. The variation in SNP loci between the *ftdm* mutant and the WT was screened to determine whether these SNPs were potential causal mutations. This screening was performed manually via Excel (version 2019), according to the following protocol: 1) SNP variation loci with a read depth ≤ 10 were filtered and removed; 2) SNPs with less than three biological replicates were filtered and removed; and 3) heterozygous SNP loci were filtered and removed. The homozygous SNPs with single-base changes between the *ftdm* mutant and the WT were used in further analysis of causal mutations.

### Analysis of differentially expressed genes (DEGs)

The threshold for corrected *p*-values was determined via the false discovery rate (FDR) (FDR < 0.001, |log2(ratio)| ≥ 1), and the criteria for identifying DEGs followed those of previously described methods, with several modifications. All the DEGs were annotated by Gene Ontology (GO) annotation terms and Kyoto Encyclopedia of Genes and Genomes (KEGG) pathways [23, 24]. The GO term and KEGG pathway analysis results were considered significant when the Bonferroni (Q-value)-corrected *p*-value was ≤0.05. The enriched KEGG pathways were determined using R software, which was also used to construct scatter diagrams of the results [25]. Furthermore, some key DEGs associated with GA synthesis according to the annotated results of the GO terms and KEGG pathways were used to construct a heatmap.

### Quantitative real-time PCR (qRT-PCR) analysis

Total RNA samples from the leaves, flowers, stems, and seeds of the *ftdm* mutant and WT plants were collected and extracted using an RNAprep Pure Kit (DP441, Tiangen Biotech, Beijing, China). Each sample included three individual biological replicates. cDNA was synthesized using a One Step PrimeScript™ RT-PCR Kit (RR064A, Takara Biomedical Technology, Shanghai, China) for qRT-PCR analysis. Gene-specific primers were designed using online software (https://www.ncbi.nlm.nih.gov/tools/primerblast/) (Table S1). The cDNA was diluted 100-fold for use as a template for qRT-PCR analysis. The total reaction volume of 20 μL consisted of 8 μL of diluted cDNA, 2 μL each of forward and reverse primers, and 10 μL of SYBR Green Real-time PCR Master Mix (FP205, Tiangen Biotech, Beijing, China); the reactions were performed within a Bio-Rad CFX96 instrument. The *histone* gene was used as a control to normalize the expression values. The relative expression level was calculated using the 2^−ΔΔCt^ method.

### Determination of endogenous phytohormone and rutin contents

For the *ftdm* and WT lines, 0.5 g of fresh stem tissue collected from N3 internodes was ground to a fine powder using liquid nitrogen with a mortar and pestle. The samples were collected from three individual biological replicates and weighed to 0.1 g for extraction. All the samples were extracted twice with 750 μL of cold extraction buffer (80% methanol:19% water:1% acetic acid (v:v:v), high-performance liquid chromatography (HPLC grade) in 1.5 mL tubes. The buffer for the first treatment was supplemented with internal standards. Plant hormones including IAA, cytokinin, GA, BRs, jasmonic acid (JA), and salicylic acid (SA) were quantified using an ultra-fast liquid chromatography-electrospray ionization tandem mass spectrometry (UFLC-ESI–MS/MS) instrument equipped with an autosampler (Waters, ACQUITY, USA), and this quantification was performed by staff at San-Shu Biotech Co. (Shanghai, China).

HPLC was used to analyse the rutin content according to Sun’s method [26]. Briefly, approximately 0.2 g of stem tissue from the WT and *ftdm* lines was frozen in liquid nitrogen and ground to a fine powder using a grinder. The powder was then added to 1 mL of methyl alcohol, macerated for 30 min, and ultrasonicated at 50 °C for extraction. The samples were centrifuged at 12,000×g for 10 min, after which the supernatant was removed and transferred to HPLC vials. HPLC analysis was performed with a C_18_ column (150 mm × 4.6 mm, 5 μm). The mobile phase consisted of methanol: H_2_O (46:54), and the retention time of rutin was 4.9 ± 0.5 min at 257 nm. The rutin content was determined using a standard curve (Fig. S1). Three biological replicates were analysed per sample, and all the samples were analysed via an Ultimate 3000 HPLC System (Thermo Scientific, USA).

### Exogenous GA treatments

Twenty-day-old seedlings were used to investigate the differences in GA responses between the WT and *ftdm* lines after treatment with GA4 combined with GA7 (G8920, Solarbio Life Sciences, Beijing, China) or treatment with H_2_O only. Three plants were sprayed with a 10 μM solution of GA4 + 7 every 7 days. The heights of the WT and *ftdm* plants were measured at 8 days, 10 days, and 14 days after spraying. An identical set of plants were sprayed with H_2_O, serving as controls.

### Sequence analysis and construction of a phylogenetic tree

The *FtSLY* (FtPinG0009116500.01) sequence was analysed via DNAMAN version 12 software. BLAST searches for homologous proteins and conserved FLY domains were performed via the National Center for Biotechnology Information (NCBI) server. Phylogenetic analysis was conducted using MEGA version 6 software based on the maximum likelihood method [27, 28].

## Results

### Comparison of the phenotypic characteristics of the *ftdm* mutant and WT plants

**Fig. 2:**
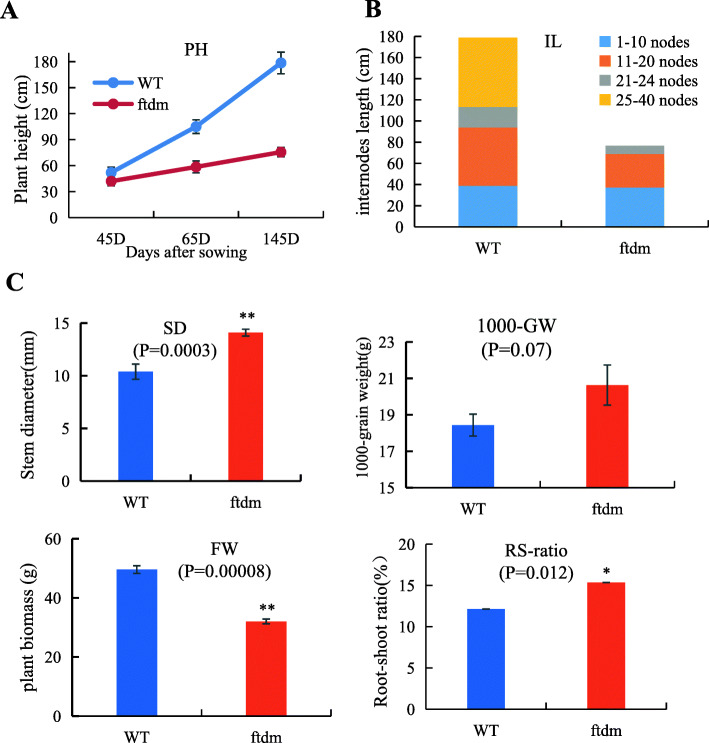
Comparisons of the main agronomic traits of the *ftdm* (dwarf mutant) and WT. **a** Change in height of the *ftdm* mutant and WT plants during the growing season. **b** Length and number of nodes of the *ftdm* mutant and WT. **c** SD, 1000-GW, FW and RS ratio of the *ftdm* mutant and WT. Note: ** indicates that the difference from the WT is extremely significant (*P* < 0.01); * indicates that the difference from the WT is significant (*P* < 0.05)

To understand the patterns of plant growth during different developmental stages, the height of the *ftdm* and WT plants was measured at three different time points. Compared with the WT plants, the *ftdm* plants grew slowly from 43 DAS to 145 DAS. At 145 DAS, the average height of the *ftdm* plants was 75.6 ± 1.98 cm, while the average height of the WT plants was 178 ± 12.77 cm. The *ftdm* mutant was approximately 42% shorter than the WT (Fig. 2a). The total length of all the stem internodes of the *ftdm* and WT plants differed in a manner similar to how the overall PH measurements differed. There were 40 main stem nodes of the WT plants; however, there were only 24 for *ftdm* plants. The length from the 11th to 20th internodes was significantly different between the *ftdm* mutant and the WT (Fig. 2b), and compared with those of the *ftdm* mutant (SD of 14.09 ± 0.33 mm), the stems of the WT (SD of 10.39 ± 0.72 mm) were significantly thinner (*P* < 0.01). The FW and RS ratio were also significantly different between the *ftdm* mutant and the WT. Surprisingly, compared with that of the WT, the 1000-GW of the *ftdm* mutant (20.6 g) was slightly higher, but this was not significantly different (Fig. 2c).

### Evaluation of cell size and lodging resistance

**Fig. 3:**
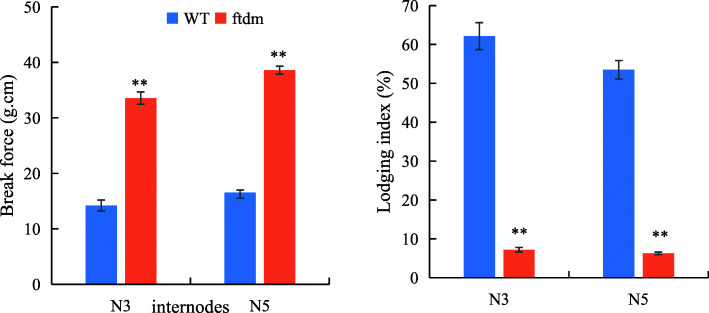
Comparisons of the BF and LI of the N3 and N5 internodes of the WT and *ftdm* mutant. ** represents a significant difference (*P* < 0.01) between the WT and *ftdm* mutant

**Fig. 4:**
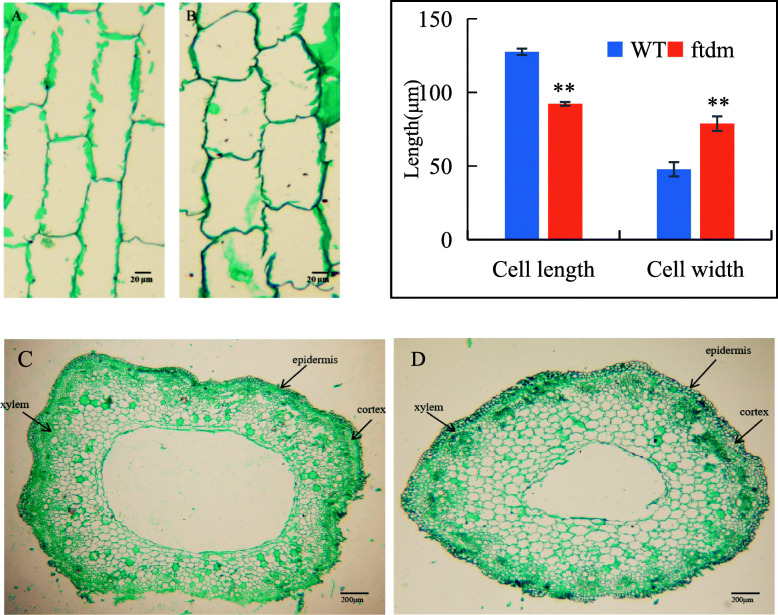
Anatomical characteristics of the N3 internode of the WT and *ftdm* mutant*.* **a** and **b** show longitudinal sections of the WT and *ftdm* mutant (magnification 100×); **c** and **d** show transverse sections of the WT and *ftdm* mutant (40×)

The LI of the *ftdm* mutant was significantly lower than that of the WT, whereas the BF of the *ftdm* mutant was higher (Fig. 3). At the N3 and N5 internodes, compared with that of the WT, the BF of the *ftdm* mutant was 150.4 and 133% higher, respectively; in contrast, the LI of the *ftdm* mutant was 88.39% (N3) and 88.27% (N5) lower. Overall, the stem cell walls of the *ftdm* mutant were thicker, the cavum was smaller, and the cell length and cell width were to some extent both shorter and wider than were those of the WT (Fig. 4). The stem cell length and width, cell wall thickness, and hollow cavity width significantly differed between the *ftdm* mutant and the WT (*P* < 0.01). The length and width of the stem cells of the WT were 127.6 ± 2.15 μm and 47.8 ± 1.33 μm, whereas those of the *ftdm* mutant were 92.2 ± 4.8 μm and 78.8 ± 4.96 μm, respectively.

### Endogenous hormone profiles, GA responses, and rutin content

**Fig. 5:**
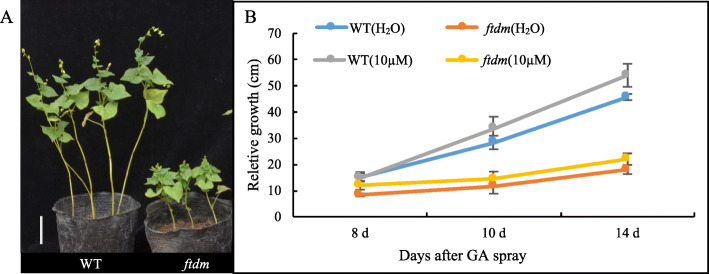
Phenotypes of the WT and *ftdm* mutant and their response to GA treatment. **a** Phenotype of WT and *ftdm* plants at 14 DAS with 10 μM GA4 + 7 every 7 days. Bar = 10 cm. **b** Relative growth of the WT and *ftdm* mutant sprayed with and without GA4 + 7

**Fig. 6:**
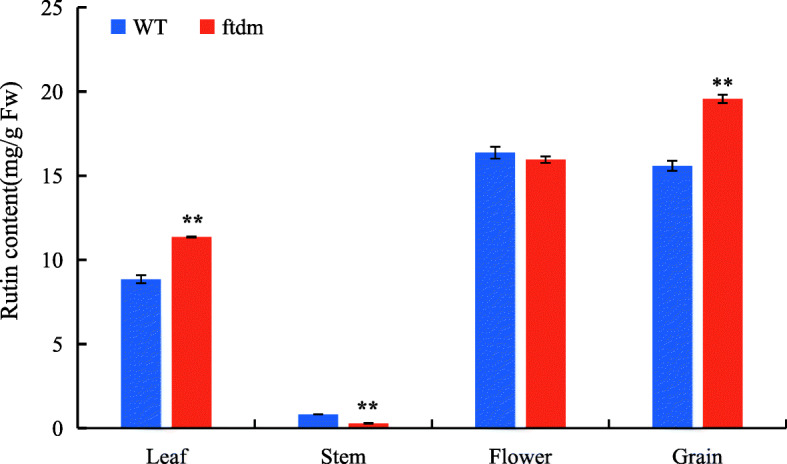
Rutin contents in the leaf, stem, flower and grain samples of WT and *ftdm* Tartary buckwheat plants. ** represents significant differences (*P* < 0.01) between the WT and *ftdm* mutant

To explore the possible role of phytohormones in PH and stem elongation of the dwarf mutant, 15 phytohormones were qualified and quantified. In terms of cytokinins, the content of trans-zeatin, 2-iP, and 2-iPA did not differ significantly between the two plant types. The zeatin content (15.20 ng/g FW) was significantly higher in the *ftdm* mutant than in the WT (*P* < 0.05). With respect to the four bioactive GAs, the contents of both GA4 (0.03 ng/g FW) in *ftdm* was decrease dramatically compare with WT (0.14 ng/g FW). And the same situation detected in GA7, the content was 0.03 ng/g FW in the *ftdm* which was significantly lower than in the WT (0.25 ng/g FW). However, the contents of JA, JA-Leu, and SA were significantly higher in the *ftdm* mutant than in the WT, and the content of methyl jasmonate (Me-JA) was significantly lower in the WT than in the *ftdm* mutant (*P* < 0.01; Table 1). Furthermore, compared with H_2_O treatment only, exogenous GA4 + 7 treatment of the *ftdm* mutant and WT caused the PH of the *ftdm* mutant to change very little. At the same stage, the WT plants were significantly taller than the mutant plants were, but they also responded very little to GA4 + 7 (Fig. 5). The rutin content was analysed in different tissues of the *ftdm* and WT plants. We found a lower concentration of rutin in the stems of the *ftdm* mutant compared with the WT, but there was no significant difference in rutin content in the floral tissue. Surprisingly, the rutin contents in the leaves and grains of the *ftdm* mutant (11.36 and 19.56 mg/g FW, respectively) were 1.28 and 1.25 times greater than those in the grains of the WT (Fig. 6).

**Table 1:** Phytohormones content in stem of HF and *ftdm*

Lines hormones	Cytokinin (CK) content (ng/g FW)				GA content (ng/g FW)				JA content (ng/g FW)						
	TZ	2-ip	2-iPA	Zeatin	GA1	GA3	GA4	GA7	JA	Me-JA	JA-leu	IAA	SA	ABA	Br
**HF**	1.09 ± 0.13	0.023 ± 0.002	0.04 ± 0.01	7.98 ± 1.09	0.39 ± 0.04	0.06 ± 0.004	0.14 ± 0.03	0.25 ± 0.07	1.69 ± 0.09	32,312.82 ± 5615.65	0.16 ± 0.02	0.09 ± 0.03	35.23 ± 1.24	31.27 ± 2.45	0.24 ± 0.14
***ftdm***	0.80 ± 0.03	0.26 ± 0.10	0.02 ± 0.01	15.20 ± 1.35	0.23 ± 0.05	0.09 ± 0.01	0.03 ± 0.002	0.03 ± 0.002	3.51 ± 0.60	117.00 ± 5.26	2.77 ± 0.08	0.09 ± 0.006	91.43 ± 0.94	31.16 ± 0.57	0.001 ± 0
***P*** **value**	0.250	0.147	0.108	0.014	0.109	0.147	0.026	0.001	0.04	0.029	0.001	0.904	0.001	0.967	0.155
**Significant**				*			*	**	*	*	**		**		

*TZ* Trans-Zeatin-riboside, *2-iP* N6-(Δ2-Isopentenyl)adenine, *2-iPA* N6-(Δ2-Isopentenyl)adenosine, *GA1* Gibberellin Acid 1, *GA3* Gibberellin Acid 3, *GA7* Gibberellin Acid 7, *IAA* 3-Indoleacetic Acid, *JA* Jasmonic acid, *JA-Leu* Jasmonic Acid-Isoleucine, *SA* salicylic acid, *ABA* Abscisic acid, *MeJA* Methyl Jasmonate, *Br* Brassinolide;

The 3rd stem from each plant at stage of harvested for the quantifification. FW, fresh weight. Data are the means SD of three independent biological samples. * indicates Signifificantly according to Tukey’s test (*P* < 0.05), while ** indicates significant (*P* < 0.01)

### Quality assessment of RNA-seq data and correlation analysis of samples

With respect to the RNA-seq data of six samples, which included the data of three biological replicates of the WT (WT-1, WT-2, and WT-3), the Q20 ranged from 96.60 to 97.68%, the minimum value of Q30 was never less than 90.81%, and the GC content ranged from 45.57 to 46.15%. The Q20 of the *ftdm* mutant (*ftdm*-1, *ftdm*-2, and *ftdm*-3) ranged from 97.55 to 97.66%, the minimum Q30 value was never less than 92.76%, and the GC content ranged from 45.73 to 45.81%, indicating that the sequencing data were of high quality (Table S2). Overall, the data indicated strong reproducibility between similar samples but revealed large differences between the WT and *ftdm* lines (Fig. S2).

### KEGG enrichment analysis of DEGs

**Fig. 7:**
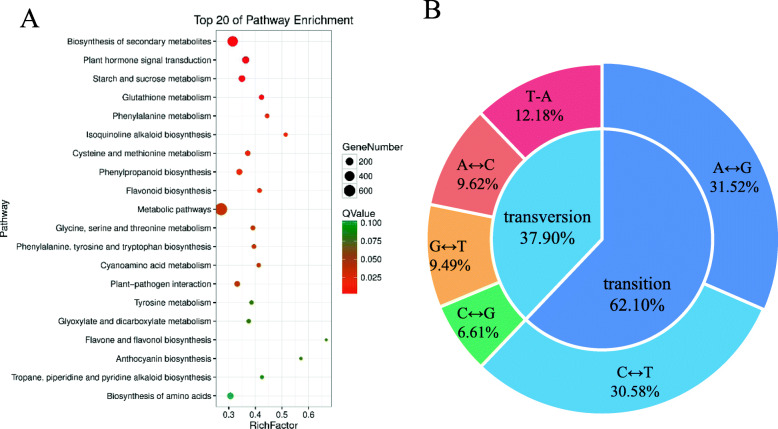
KEGG enrichment analysis and SNP type analysis. **a** Scatter diagram of the top 20 statistically enriched pathways according to KEGG pathway analysis of *ftdm* and WT plants. **b** Statistics of SNP mutation types

To explore potential differences in gene expression related to specific biological pathways on the basis of the RNA-seq data between the *ftdm* mutant and the WT, 1614 DEGs were identified and mapped to 129 KEGG pathways. Twenty KEGG pathways were significantly enriched (Fig. 7a) and are listed in Table S3. The first major enriched KEGG pathway was biosynthesis of secondary metabolites, which involved 454 DEGs, accounting for 28.13% of the total DEGs. The next most enriched KEGG pathways were plant hormone signal transduction, starch and sucrose metabolism, phenylpropanoid biosynthesis, and amino acid biosynthesis, accounting for 8.67, 7.06, 5.27, and 6.01% of the total DEGs, respectively.

### SNP loci identified from comparison of transcriptomic data

**Fig. 8:**
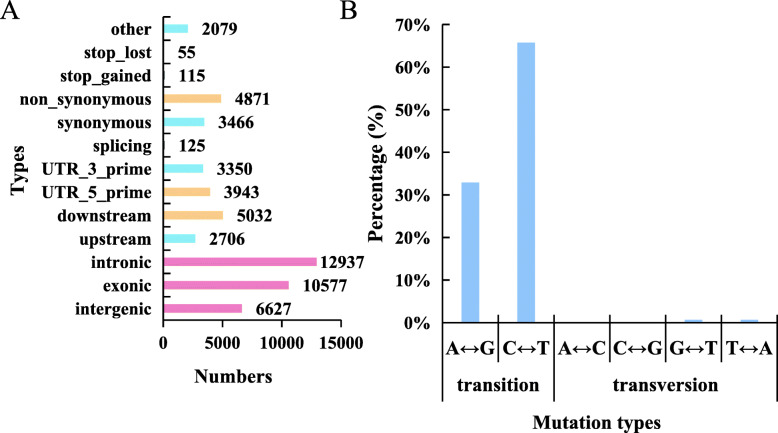
Functional classification, location and types of SNPs. **a** SNP function classification and location statistics. **b** Percentage of SNP mutation types

In total, 28,008 SNP loci were identified by comparisons of the RNA-seq data and the reference genome. Specifically, 17,393 SNP transition sites and 10,615 SNP transversion sites were detected, accounting for 62.1 and 37.9%, respectively, of the total SNPs. The number of SNP transition sites was 1.63-fold greater than the number of transversion sites (Fig. 7b). Additionally, among these SNP variations, the proportion of C-to-G transitions was the lowest, accounting for only 6.61%. In total, 12,937 SNPs were located in intron regions, accounting for 28.12%, the frequency of which was significantly higher than for the other types. In addition, 10,577, 6627, and 5032 SNPs were located in the exons, intergenic regions, and downstream regions of genes, respectively. Among these SNPs, 4871 nonsynonymous and 3466 synonymous mutations were detected (Fig. 8a).

Finally, through manual filtering of the detected SNPs, mutations of 146 homozygous SNP loci were identified by comparisons between the *ftdm* mutant and the WT. SNP transition sites accounted for 98.63% of the total. There were 48 A-to-G and 96 C-to-T conversions, accounting for 32.88 and 65.75%, respectively, of the total homozygous SNPs. Additionally, the remaining 1.37% of SNP loci comprised G-to-T and T-to-A transition mutations. No transitions involving A to C or C to G were detected (Fig. 8b). These homozygous SNPs involved 101 nonsynonymous mutations, three stop codons, and 42 synonymous mutations.

### Prediction and identification of candidate genes related to the dwarf trait

**Fig. 9:**
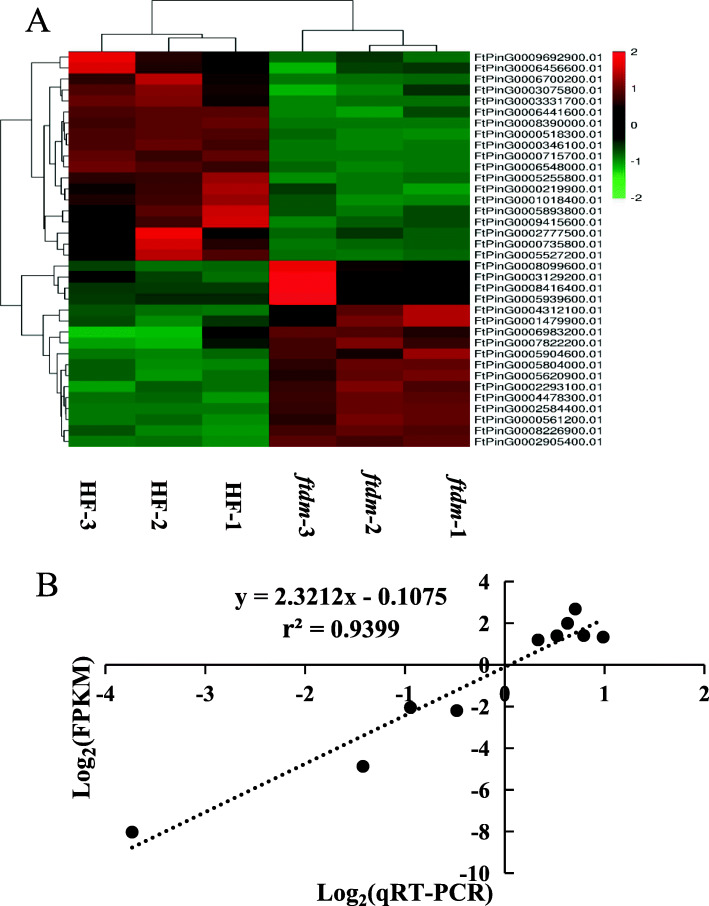
Heatmap of DEGs (**a**) and qRT-PCR verification analysis of the RNA-seq sequencing data (**b**) from the WT and *ftdm* mutant

To identify candidate genes related to the dwarf trait, homozygous SNPs involving nonsynonymous mutations were evaluated to analyse their effects on differential expression between the *ftdm* mutant and the WT. Twelve DEGs with nonsynonymous SNPs were found, eight of whose expression was downregulated and four of whose expression was upregulated in the *ftdm* mutant but not in the WT (Fig. 9a). The mutation types in these DEGs included nine C-to-T and three G-to-A transitions. GO functional annotation of these DEGs showed that there were seven genes involved in molecular functions, three genes involved in cellular components, and one gene involved in biological processes. These genes were named GPAT8, WAT1, SKIP14, PUB30, CIPK14, MED15A, CHUP1, STR4, SS1, ZFP1, ABCG21, and UGT92J1, and their homology/function was predicted on the basis of UniProt protein annotation information (Table S4).

The expression levels of these 12 candidate genes in the stem tissues of the *ftdm* mutant and the WT, as determined via qRT-PCR, were strongly correlated with the results of the RNA-seq analysis (R^2^ = 0.9399) and the DEG analysis (Fig. 9b).

**Fig. 10:**
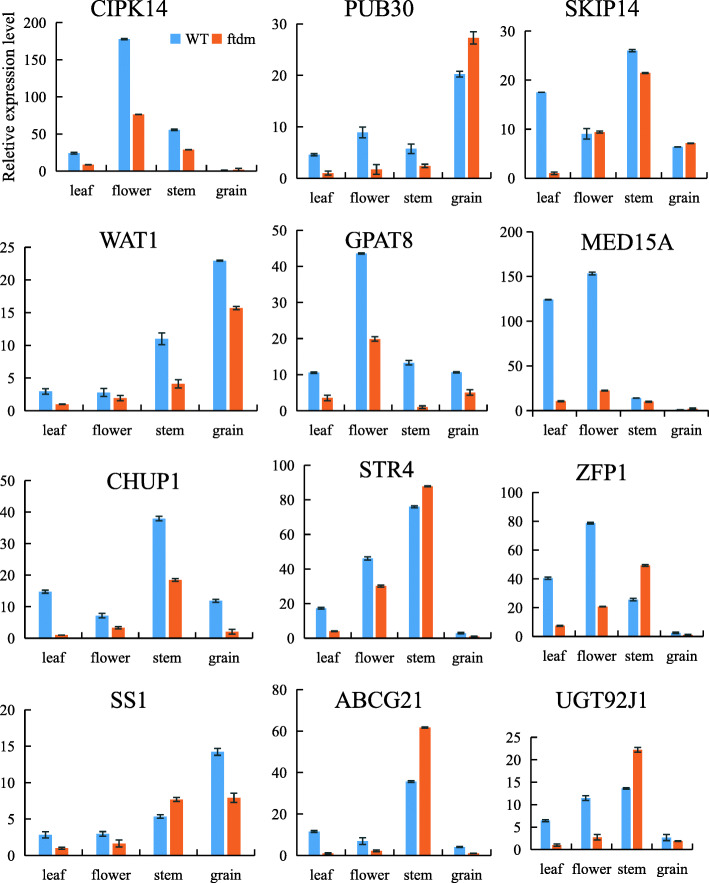
Expression of select genes in different WT and *ftdm* tissues, as measured *via* qRT-PCR. CIPK 14: CBL-interacting serine/threonine-protein kinase 14; PUB 30: U-box domain-containing protein 30-like; SKIP14: F-box protein, SKIP14; WAT1: WALLS ARE THIN 1-like; GPAT8: glycerol-3-phosphate acyltransferase 8; MED15A: Mediator of RNA polymerase II transcription subunit 15; CHUP1: protein CHUP1, chloroplastic isoform X1; STR4: rhodanese-like domain-containing protein 4; ZFP1: Zinc finger CCCH domain-containing protein 1; SS1: starch synthase 1; ABCG21: ABC_tran domain-containing protein/ABC2_membrane domain-containing protein; UGT92J1: glycosyltransferase UGT92J1

The tissue-specific expression of SNPs in the leaves, floral tissue, stems, and grains of the *ftdm* mutant and the WT were further analysed. The data showed that the expression levels of the SKIP14, CHUP1, STR4, ABCG21, and UGT92J1 genes in the stems were significantly higher than those in other tissues. The expression levels of CIPK14, GPAT8, ZFP1, and MED15A were higher in the floral organs than in the other organs. Similarly, the expression levels of PUB30, WAT1, and SS1 in the grains were higher than those in the other tissues (Fig. 10). We noticed that the SKIP14-specific expression in the stems and the relative expression levels in the stems of the *ftdm* mutant were 4.48 times lower than those in the WT.

### SKIP14 sequence characteristics and expression patterns of genes related to the GA synthesis pathway

**Fig. 11:**
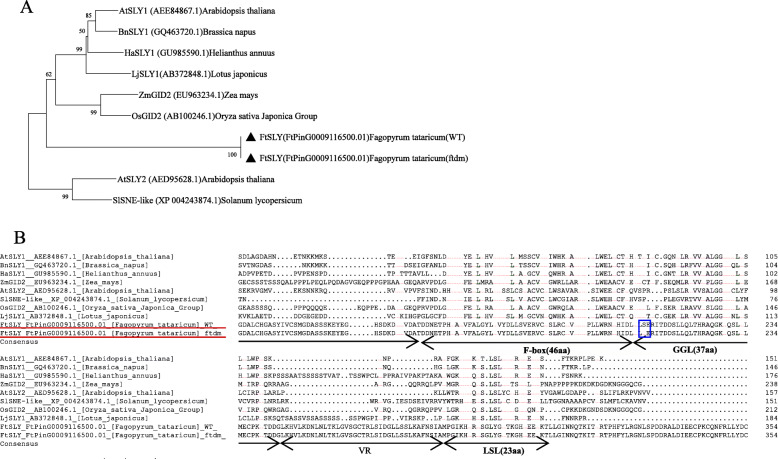
Sequence and expression analysis of *FtSLY* in the WT and *ftdm* mutant. **a** Phylogenetic analysis of *FtSLY* and other F-box proteins was performed by the neighbour-joining method in MEGA version 6. The values above the branches are 500 bootstrap percentage analyses of 1000 replicates. **b** Multiple sequence alignment of *FtSLY* and other F-box proteins. Identical amino acids are shaded in black, and similar amino acids are shaded in green. GGL and LSL refer to conserved residues. aa: amino acids; VR, variable region. The black box shows the amino acid change from Ser (S) to Asn (N)

We detected an amino acid mutation involving a change of Ser in the WT to Asn in the *ftdm* mutant located within the GGL conserved domain of SKIP14. Using phylogenetic analysis, we found that SKIP14 (FtPinG0009116500.01) clustered together with AtSLY1, BnSLY1, HaSLY1, LjSLY1, ZmGID2, and OsGID2 on the basis of their homology (Fig. 11a). The deduced protein sequence of SKIP14 revealed similar conserved domains, including F-box, GGL, and LSL domains, that are homologous to those of related proteins in other species (Fig. 11b). These results suggest that SKIP14 in Tartary buckwheat encodes an F-box protein that is a component of the ASK-cullin-F-box (SCF) E3 ubiquitin ligase complex and may have a function similar to that of SLY1 in Arabidopsis. Based on sequence homology, this gene may be responsible for ubiquitination and subsequent proteasomal degradation of DELLA proteins, controlling GA response signalling.

**Fig. 12:**
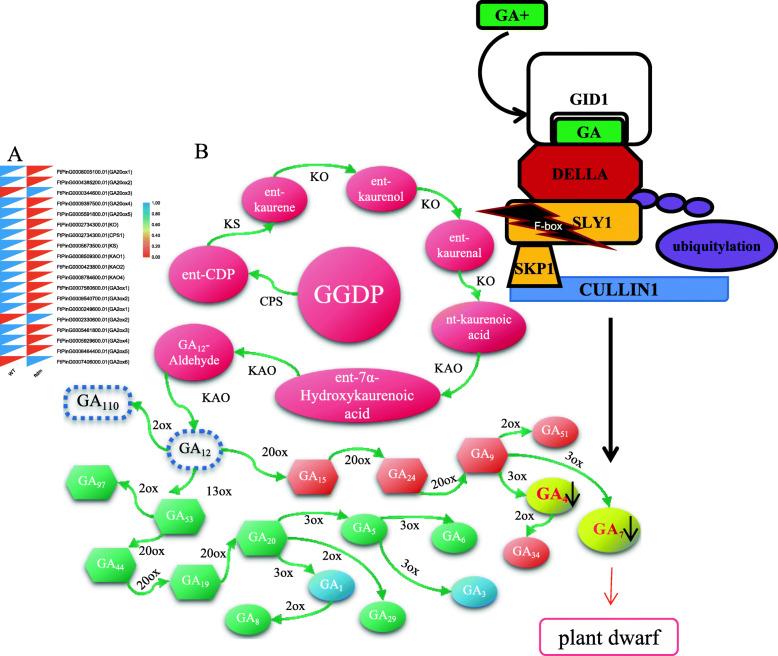
Expression level of GA biosynthesis pathway-related genes (**a**) and a model of the F-box gene regulation of dwarfness of the *ftdm* mutant (**b**). GGDP: trans-geranylgeranyl diphosphate; CPS: ent-copalyldiphosphate synthase; KS: ent-kaurene synthase; KO: ent-kaurene oxidase; KAO: entkaurenoic acid oxidase; 20ox: GA 20-oxidase; 3ox: GA 3-oxidase; 2ox: GA 2-oxidase; GID: gibberellin receptor protein; SLY: SLEEPY. The black arrow indicates a decrease in the contents of GA4 and GA7. The black asterisk indicates the mutation detected in the F-box protein SKIP14

Nineteen genes involved in the GA synthesis pathway were identified from the transcriptomic data, and 16 of them were expressed at lower levels in the stems of the *ftdm* mutant compared to the WT (Fig. 12a). The lower expression levels of these genes could result in a reduction in GA4 + GA7 content in the stems of the *ftdm* mutant. Furthermore, a coordinated expression trend was observed for SKIP14 and GA biosynthesis-related genes in the *ftdm* mutant (Fig. 12b).

## Discussion

### Evaluation of lodging resistance of the *ftdm* mutant and the WT

We identified a Tartary buckwheat dwarf mutant by making selections from an EMS-mutagenized population. During the last century, the successful application of dwarf and semi-dwarf wheat and rice mutants has greatly increased crop yields, improving the food supply in Asia [12, 29–31]. With the increase in population and reduction in available arable land, there has been an increased focus on minor crop species to improve the diversity of the food supply to address climate change and food crises. Tartary buckwheat is an important minor crop species that is commonly used to alleviate food disasters in China. Despite the importance of Tartary buckwheat, very few studies have been conducted with the goal of improving yield through reductions in PH.

PH, SD, stem BF, and crop biomass have been shown to be closely related to lodging resistance [32, 33]. Due to the height and branching characteristics of Tartary buckwheat, lodging is a very severe problem during harvest, preventing the economic development of this crop species. Shimizu identified two semi-dwarf genes (sdA and sdB) in Tartary buckwheat [34] and developed the semi-dwarf cultivar Darumadattan, whose height is controlled by the single nuclear recessive gene sdA [35]. We evaluated the lodging resistance of the dwarf mutant *ftdm*, which was derived from EMS mutagenesis. Previous studies have assessed the lodging resistance of four genotypes of Tartary buckwheat; the results showed that the LI was significantly negatively correlated with lignin content, BF, and number of vascular bundles [9]. Another study revealed that lodging resistance was significantly correlated with both SD and stem length [36]. Our results showed that the dwarf mutant had a higher lodging resistance than the WT did, mostly because of the 42% reduction in height and increased SD of the former (Figs. 2, 3). On the basis of cytological evidence, we found that the shorter and thicker stems in the *ftdm* mutant compared with the WT were due to larger cells in that organ. Zhou [37] investigated the main agronomic traits of 371 Tartary buckwheat accessions and found that height, 1000-GW, and grain weight per plant were important components influencing yield. Our results indicated that a shorter height and thicker SD can increase the resistance of Tartary buckwheat to lodging. However, compared with the WT, the *ftdm* mutant did not have a significantly lower 1000-GW or other major yield traits. Given these results, we speculated that, compared with the WT, the *ftdm* mutant may present increased yield potential and is suitable for mechanized harvesting.

### *ftdm* is a GA-insensitive dwarf mutant with increased rutin content

Phytohormones are key regulators that affect plant growth, development, and responses to stress [38]. GAs, including GA1, GA3, GA4, and GA7, comprise a large hormone group and have been detected at different stages of rice development [39]. The current molecular model of GA signal transduction can be summarized as follows: in the absence of GA synthesis, DELLA proteins accumulate and inhibit the GA signal response, thus inhibiting plant growth; under the conditions of sufficient GA synthesis or exogenous GA induction, the GA binding receptor GID1 stimulates the formation of the GA-GID1-DELLA protein complex, after which the F-box protein ubiquitinates the complex. DELLA proteins are subsequently degraded through the 26S proteasome to release the growth inhibition regulated by the DELLA proteins and initiate the GA signal transduction response [40]. The latest research shows that through clustered, regularly interspaced, short palindromic repeat (CRISPR)/CRISPR-associated 9 (Cas9) technology, editing the active domain of tomato DELLA proteins can enhance the stability of DELLA proteins; the degradation of DELLA proteins then becomes blocked, resulting in GA-insensitive function dwarf mutants and serving as a means to artificially create dwarf mutants [41]. In our study, in terms of GAs, the contents of only GA4 and GA7 were significantly lower in the dwarf mutant than in the WT. Treating the *ftdm* mutant with exogenous GAs did not restore the WT PH phenotype. These results indicate that the *ftdm* mutant is unable to respond strongly to GA4 and GA7*.* However, this does not explain the lower expression of GA biosynthesis-related genes (Fig. 12) or the lower GA4 and GA7 content in the stems of the *ftdm* mutant compared with the WT (Table 1). Furthermore, compared with those of the WT, the phytohormone profiles of the stems of the *ftdm* mutant revealed significantly higher contents of zeatin, JAs (except Me-JA), and SA. Taken together, these findings might best be explained by altered hormone signalling and feedback regulation in the mutant affecting more than one hormone. The higher zeatin, JA, and SA contents in the mutant could potentially upregulate the expression of other genes, such as those involved in secondary metabolism and responses to stress.

Previously, we found that the rutin content in the leaves of Tartary buckwheat could be increased by SA and Me-JA treatment [26, 42]. Surprisingly, the rutin contents in the leaves and grains of Tartary buckwheat were significantly higher in the *ftdm* mutant than in the WT. A possible explanation is that the increased endogenous SA content in the stem could induce rutin synthesis, which would then be transported to the leaves and grains through the vascular tissue. BR was absent from the stems of the *ftdm* mutant, which could explain the decreased height and decreased number branches in the dwarf mutant. Zhu [43] found that transgenic tomato plants whose BR receptor gene was silenced presented reduced BR contents and exhibited dwarf phenotypes. It is also important to consider cross-talk between different hormones; specifically, inactivation of DELLA proteins, the regulator of GAs, represses BR receptors, regulating the BR content in vivo [44].

### F-box proteins may be key candidate genes associated with the dwarf phenotype of the *ftdm* mutant

According to our recent research, a hybrid population of the *ftdm* mutant (♀) and Heifeng No. 1 (♂) was generated. The progeny separation ratio of the F2 generation was investigated, which was found to be consistent with the classic 3:1 mendelian ratio. Therefore, we speculated that the gene that causes plant dwarfing may be controlled by a recessive single gene (unpublished data). We found that the expression levels of 16 genes involved in the GA synthesis pathway were significantly lower in the *ftdm* mutant than in the WT. The expression patterns of these genes were consistent with the results concerning GA deficiency. However, we did not find any nucleotide sequence mutations in these genes via transcriptomic sequencing analysis. We searched for indications of possible losses of functions of genes that are related to height and GA signalling and that could be related to the dwarf phenotype*.* By performing SNP variant analysis, we identified 12 genes with nonsynonymous mutations and found that some genes were expressed at a lower level in the stems of the *ftdm* mutant compared with the WT. WAT1, SKIP14, and UGT92J1 were expressed specifically in the stems of the *ftdm* mutant, but only SKIP14 was expressed at a level lower in the mutant than in the WT. Compared with WT Arabidopsis, the Arabidopsis *wat1* mutant has shorter stems and accumulates higher amounts of flavonols and flavonol glycosides because the *WAT1* gene is responsible for controlling lignin and flavonoid synthesis and secondary wall development in fibres [45]. SKIP14 encodes an F-box protein that is highly homologous to SLY1 in Arabidopsis. SKIP14 is a component of the SCF E3 ubiquitin ligase complex, which might mediate the ubiquitination and subsequent degradation of DELLA proteins. The SNP detected in SKIP14 may suggest that DELLA proteins cannot be degraded; this phenomenon could repress GA synthesis or signalling in vivo and might result in a GA-insensitive dwarf phenotype. Hence, we speculated that SKIP14 may be a key candidate gene regulating the GA response in the *ftdm* mutant, although at present, there is no direct evidence that the mutation in SKIP14 alters its function.

It is also possible that more than one gene is responsible for the dwarf phenotype. For example, the higher rutin and flavonol glycosides that accumulated in the *ftdm* mutant compared with the WT may also be associated with downregulated WAT1 expression. ABCG21 is involved in both IAA transport and the environmental stress response [46] and acts as an important regulator of growth and development of plant stems [47]. A previous report showed that CIPK14 interacts with calcineurin B-like protein (CBLs) and controls glucose signalling in Arabidopsis. In tobacco, overexpression of the AtZFP10 gene resulted in a dwarf phenotype, an abnormal leaf phenotype, and early flowering. In our study, the expression level of ZFP1, which was highly homologous to AtZFP10, was upregulated in the stems of the *ftdm* mutant, the phenomenon of which might be associated with the dwarf phenotype. Future studies are needed to determine the major gene controlling the dwarf trait, and verification of the function of this gene by quantitative trait locus (QTL) analysis and genetic engineering is also needed.

## Conclusion

A dwarf mutant line of Tartary buckwheat was identified by screening an EMS mutant population. Several differentially expressed candidate dwarf genes with point mutations were detected, including a single amino acid mutation in the F-box domain of FtSKIP14. The endogenous GA4 + 7 content significant decreased in the *ftdm* mutant compared with the WT*.* This study was the first to generate a dwarf line and study the mechanism underlying dwarfism in Tartary buckwheat.

## Supplementary Information

**Additional file 1: Fig. S1.** Peak area (A) and standard curve (B) of rutin, as determined via HPLC.

**Additional file 2: Fig. S2.** Heat map showing inter-sample correlations between the WT and *ftdm* mutant. All the samples involved three biological replication.

**Additional file 3.**

**Additional file 4.**

**Additional file 5.**

**Additional file 6.**

## Data Availability

All datasets supporting the results of this article are included within the article and its supplementary information.
