# Correction to: Comparative transcriptomic analysis reveals the regulatory mechanism of the gibberellic acid pathway of Tartary buckwheat (*Fagopyrum tataricum* (L.) Gaertn.) dwarf mutants

**DOI:** 10.1186/s12870-021-03014-5

**Published:** 2021-05-27

**Authors:** Zhaoxia Sun, Xinfang Wang, Ronghua Liu, Wei Dun, Mingchuan Ma, Yuanhuai Han, Hongying Li, Longlong Liu, Siyu Hou

**Affiliations:** 1 College of Agriculture, Institute of Agricultural Bioengineering, Shanxi Agricultural University, Shanxi, 030801 China; 2 Key Laboratory of Crop Gene Resources and Germplasm Enhancement on Loess Plateau, Ministry of Agriculture, Shanxi, 030031 China; 3 Center for Agricultural Genetic Resources Research, Shanxi Agricultural University, Shanxi, 030031 China; 4 Shanxi Key Laboratory of Minor Crop Germplasm Innovation and Molecular Breeding, Shanxi, 030031 China

**Correction to: BMC Plant Biol 21, 206 (2021)**

**https://doi.org/10.1186/s12870-021-02978-8**

Following publication of the original article [1], the authors identified an error in the author’s affiliations. Zhaoxia Sun, Siyu Hou, and Yuanhuai Han are affiliated to:

- ^3^Shanxi Key Laboratory of Minor Crop Germplasm Innovation and Molecular Breeding, Taiyuan, 030031, Shanxi, China

Mingchuan Ma and Longlong Liu are affiliated to:

- ^4^Center for Agricultural Genetic Resources Research, Shanxi Agricultural University, Taiyuan, 030031, Shanxi, China
  The original article has been corrected.
