# Supplementary figures and images for: Comparative transcriptomic analysis reveals the regulatory mechanism of the gibberellic acid pathway of Tartary buckwheat (Fagopyrum tataricum (L.) Gaertn.) dwarf mutants
Source: BMC Plant Biol. 2021 Apr 30;21:206. doi: 10.1186/s12870-021-02978-8 (PMC8086092; doi:10.1186/s12870-021-02978-8)

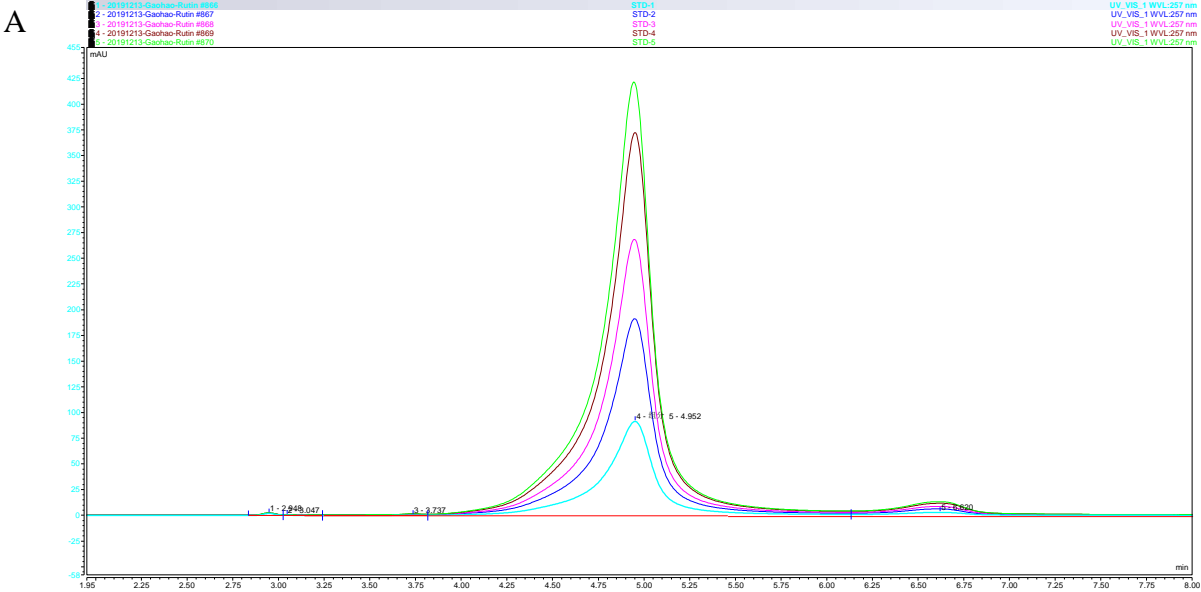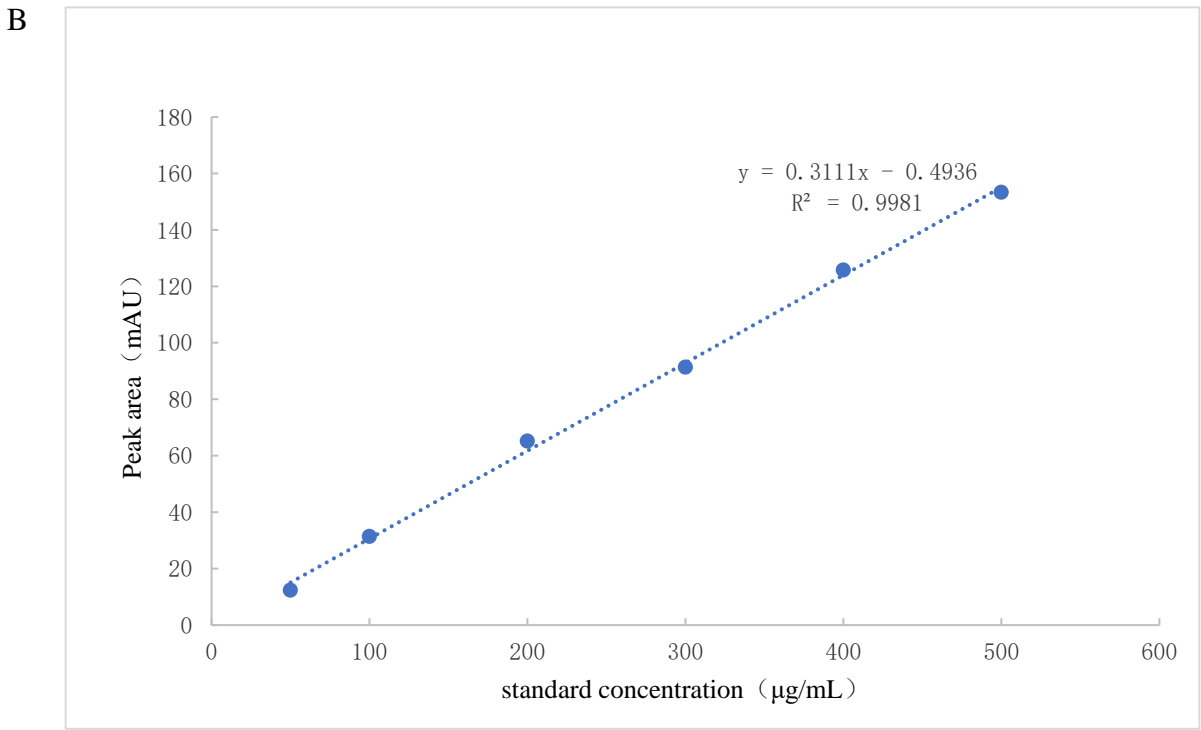

Fig. S1 Peak area (A) and standard curve (B) of rutin, as determined via HPLC.

Supplement: Supplementary file 1 — Additional file 1: Fig. S1. Peak area (A) and standard curve (B) of rutin, as determined via HPLC. [file 12870_2021_2978_MOESM1_ESM.pdf]
