# Supplementary material for: Comparative transcriptomic analysis reveals the regulatory mechanism of the gibberellic acid pathway of Tartary buckwheat (Fagopyrum tataricum (L.) Gaertn.) dwarf mutants
Source: BMC Plant Biol. 2021 Apr 30;21:206. doi: 10.1186/s12870-021-02978-8 (PMC8086092; doi:10.1186/s12870-021-02978-8)
Supplement: Supplementary file 2 — Additional file 2: Fig. S2. Heat map showing inter-sample correlations between the WT and ftdm mutant. All the samples involved three biological replication. [file 12870_2021_2978_MOESM2_ESM.pdf]

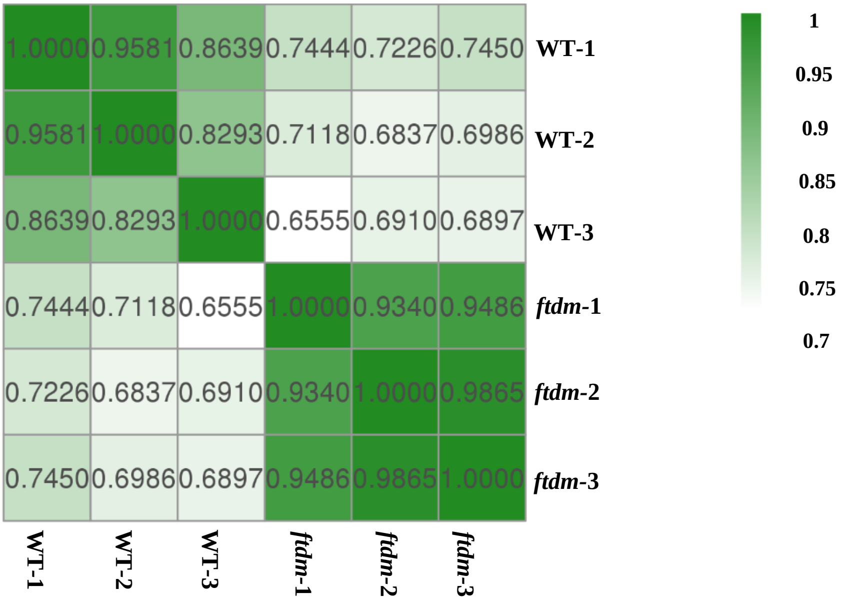

Fig. S2 Heat map showing inter-sample correlations between the WT and *ftdm* mutant. All the samples involved three biological replication.
