# Supplementary material for: Comparative transcriptomic analysis reveals the regulatory mechanism of the gibberellic acid pathway of Tartary buckwheat (Fagopyrum tataricum (L.) Gaertn.) dwarf mutants
Source: BMC Plant Biol. 2021 Apr 30;21:206. doi: 10.1186/s12870-021-02978-8 (PMC8086092; doi:10.1186/s12870-021-02978-8)
Supplement: Supplementary file 3 — Additional file 3. [file 12870_2021_2978_MOESM3_ESM.docx]

Table S1 Primers used for q-RT PCR

| Primer name | Sequences (5´-3´) |
| --- | --- |
| CIPK14 | F: CCGGACACGAGGATCACAAT |
|  | R: TGCGTTCATGGTTCTGCTCT |
| PUB30 | F: GTTGGTTCTGAGGCTGTATGTATT |
|  | R: TGGACGCCATTGGAGTGT |
| SKIP14 | F: TTGGGATTTGGTTCTTACTCG |
|  | R: GCATCCACTTCCTCGTCTTTA |
| WAT1 | F: TCGTCACTCGAAACGAACCA |
|  | R: TCCACCAAACAGGATGGCTG |
| GPAT8 | F: TCTACTTCAACATCCCTTTACCG |
|  | R: ATCATCTCGGCGTCAGCG |
| MED15A | F: CAAGGCAGCTTCCACATGTT |
|  | R: ATATTCGCCGGAGATAATCAA |
| CHUP1 | F: GCGGCTTGTAGTTACCGTGATA |
|  | R: TACCGCTTACTCGTACCGTCTC |
| STR4 | F: GGTGACTGCCAATGGGTTC |
|  | R: TGAATGCCAACACTCCAAGC |
| ZFP1 | F: TTCCAAGAGCACAATGTTTCACTA |
|  | R: TATGAGCAGATGCCCTAAGTGG |
| SS1 | F: GAGTGGACCAGGAAACAAAGAT |
|  | R: AGGTGCTGCCTCTGAAGTGA |
| ABCG21 | F: GGCTCCATAACATACAACGACC |
|  | R: GTTTACCAGCATTTCTTGACCTAT |
| UGT92J1 | F: TTCAGAAGGAAGCTCCAGCG |
|  | R: GGCGACTTACTGTCAAGCCA |
| His-Q | F: ATCGACTGGAGGAAAGGCTC |
|  | R: GCGGTATCTGTGGGACTTCT |
