# Supplementary material for: Comparative transcriptomic analysis reveals the regulatory mechanism of the gibberellic acid pathway of Tartary buckwheat (Fagopyrum tataricum (L.) Gaertn.) dwarf mutants
Source: BMC Plant Biol. 2021 Apr 30;21:206. doi: 10.1186/s12870-021-02978-8 (PMC8086092; doi:10.1186/s12870-021-02978-8)
Supplement: Supplementary file 4 — Additional file 4. [file 12870_2021_2978_MOESM4_ESM.docx]

Table S2 Filtered data quality assessment comparing WT and *ftdm*.

| Sample Name | Clean reads | High quality clean reads | Q20(%) | Q30(%) | GC content(%) |
| --- | --- | --- | --- | --- | --- |
| WT-1 | 7737859224 | 7274645764 | 7099362874 (97.59%) | 6764800054 (92.99%) | 3317034699 (45.60%) |
| WT-2 | 7307718648 | 6876479154 | 6716759325 (97.68%) | 6409828323 (93.21%) | 3133340103 (45.57%) |
| WT-3 | 7642284090 | 7049452150 | 6809732388 (96.60%) | 6401839809 (90.81%) | 3253173840 (46.15%) |
| *ftdm*-1 | 7601057400 | 7330103645 | 7155622894 (97.62%) | 6809242623 (92.89%) | 3357624755 (45.81%) |
| *ftdm*-2 | 9315729600 | 8972479272 | 8752502699 (97.55%) | 8323109833 (92.76%) | 4104188425 (45.74%) |
| *ftdm*-3 | 7625863200 | 7361607473 | 7189070112 (97.66%) | 6847875323 (93.02%) | 3366752676 (45.73%) |
