# Supplementary material for: Comparative transcriptomic analysis reveals the regulatory mechanism of the gibberellic acid pathway of Tartary buckwheat (Fagopyrum tataricum (L.) Gaertn.) dwarf mutants
Source: BMC Plant Biol. 2021 Apr 30;21:206. doi: 10.1186/s12870-021-02978-8 (PMC8086092; doi:10.1186/s12870-021-02978-8)
Supplement: Supplementary file 5 — Additional file 5. [file 12870_2021_2978_MOESM5_ESM.docx]

Table S3 Analysis of KEGG pathways for DEGs between *ftdm1* and WT

| Pathway | DEGs genes with pathway annotation | All genes with pathway annotation | *P* value | Pathway ID |
| --- | --- | --- | --- | --- |
| Biosynthesis of secondary metabolites | 454 (28.13%) | 1442 (22.62%) | 0.000000 | ko01110 |
| Plant hormone signal transduction | 140 (8.67%) | 385 (6.04%) | 0.000000 | ko04075 |
| Starch and sucrose metabolism | 114 (7.06%) | 326 (5.11%) | 0.000043 | ko00500 |
| Glutathione alkaloid biosynthesis | 47 (2.91%) | 111 (1.74%) | 0.000058 | ko00480 |
| Phenylalanine metabolism | 28 (1.73%) | 63 (0.99%) | 0.000706 | ko00360 |
| Isoquinoline alkaloid biosynthesis | 18 (1.12%) | 35 (0.55%) | 0.000791 | ko00950 |
| Cysteine and methionine metabolism | 55 (3.41%) | 148 (2.32%) | 0.000838 | ko00270 |
| Phenylpropanoid biosynthesis | 85 (5.27%) | 250 (3.92%) | 0.001098 | ko00940 |
| Flavonoid biosynthesis | 32 (1.98%) | 77 (1.21%) | 0.001246 | ko00941 |
| Metabolic pathways | 713 (44.18%) | 2627 (41.21%) | 0.002811 | ko01100 |
| Glycine, serine and threonine metabolism | 34 (2.11%) | 87 (1.36%) | 0.003076 | ko00260 |
| Phenylalanine, tyrosine and tryptophan biosynthesis | 32 (1.98%) | 81 (1.27%) | 0.003297 | ko00400 |
| Cyanoamino acid metabolism | 26 (1.61%) | 63 (0.99%) | 0.003870 | ko00460 |
| Plant-pathogen interaction | 75 (4.65%) | 226 (3.55%) | 0.004289 | ko04626 |
| Tyrosine metabolism | 27 (1.67%) | 70 (1.1%) | 0.009596 | ko00350 |
| Glyoxylate and dicarboxylate metabolism | 30 (1.86%) | 80 (1.25%) | 0.010309 | ko00630 |
| Flavone and flavonol biosynthesis | 6 (0.37%) | 9 (0.14%) | 0.010620 | ko00944 |
| Anthocyanin biosynthesis | 8 (0.5%) | 14 (0.22%) | 0.011084 | ko00942 |
| Tropane, piperidine and pyridine alkaloid biosynthesis | 17 (1.05%) | 40 (0.63%) | 0.012885 | ko00960 |
| Biosynthesis of amino acids | 97 (6.01%) | 317 (4.97%) | 0.016987 | ko01230 |
