# Supplementary material for: Comparative transcriptomic analysis reveals the regulatory mechanism of the gibberellic acid pathway of Tartary buckwheat (Fagopyrum tataricum (L.) Gaertn.) dwarf mutants
Source: BMC Plant Biol. 2021 Apr 30;21:206. doi: 10.1186/s12870-021-02978-8 (PMC8086092; doi:10.1186/s12870-021-02978-8)
Supplement: Supplementary file 6 — Additional file 6. [file 12870_2021_2978_MOESM6_ESM.docx]

| Gene ID | Chr. | SNP | Log2(FC) | Symbol | Description | GO |
| --- | --- | --- | --- | --- | --- | --- |
| FtPinG0000346100.01 | Ft6 | C→T | -5.08 | GPAT8 | glycerol-3-phosphate acyltransferase | Molecular Function: 1-acylglycerol-3-phosphate O-acyltransferase activity (GO:0003841) |
| FtPinG0006548000.01 | Ft1 | C→T | -4.87 | WAT1 | PREDICTED: protein WALLS ARE THIN 1-like | Biological Process: polysaccharide biosynthetic process (GO:0000271) |
| FtPinG0009116500.01 | Ft3 | C→T | -2.38 | SKIP14 | F-box protein SKIP14 GN=SKIP14 | -- |
| FtPinG0002777500.01 | Ft7 | C→T | -2.08 | PUB30 | PREDICTED: U-box domain-containing protein 30-like | Cellular Component: ubiquitin ligase complex (GO:0000151) |
| FtPinG0001018400.01 | Ft1 | C→T | -2.05 | CIPK14 | PREDICTED: CBL-interacting serine/threonine-protein kinase 14 | Molecular Function: calmodulin-dependent protein kinase activity (GO:0004683) |
| FtPinG0000219900.01 | Ft2 | G→A | -1.28 | MED15A | Mediator of RNA polymerase II transcription subunit 15 | Molecular Function: kinase activity (GO:0016301) |
| FtPinG0006456600.01 | Ft1 | C→T | -1.25 | CHUP1 | PREDICTED: protein CHUP1, chloroplastic isoform X1 | Cellular Component: chloroplast envelope (GO:0009941) |
| FtPinG0000715700.01 | Ft2 | C→T | -1.10 | STR4 | PREDICTED: rhodanese-like domain-containing protein 4 | Cellular Component: chloroplast (GO:0009507) |
| FtPinG0005939600.01 | Ft7 | C→T | 1.22 | SS1 | PREDICTED: starch synthase 1, chloroplastic/amyloplastic | Molecular Function: starch synthase activity (GO:0009011) |
| FtPinG0002584400.01 | Ft2 | G→A | 1.40 | ZFP1 | Zinc finger CCCH domain-containing protein 1 | Molecular Function: nucleic acid binding (GO:0003676) |
| FtPinG0003129200.01 | Ft5 | C→T | 1.41 | ABCG21 | ABC_tran domain-containing protein | Molecular Function: ATP binding (GO:0005524) |
| FtPinG0005904600.01 | Ft6 | G→A | 2.68 | UGT92J1 | UDP-glycose: glycosyltransferase UGT92J1 [Fagopyrum esculentum] | Molecular Function: transferase activity, transferring glycosyl groups (GO:0016757) |

Table S4 The annotation information of candidate genes related to *ftdm* trait
